# A novel fatty acid metabolism-related signature identifies features of the tumor microenvironment and predicts clinical outcome in acute myeloid leukemia

**DOI:** 10.1186/s12944-022-01687-x

**Published:** 2022-08-25

**Authors:** Hai-Bin Zhang, Zhuo-Kai Sun, Fang-Min Zhong, Fang-Yi Yao, Jing Liu, Jing Zhang, Nan Zhang, Jin Lin, Shu-Qi Li, Mei-Yong Li, Jun-Yao Jiang, Ying Cheng, Shuai Xu, Xue-Xin Cheng, Bo Huang, Xiao-Zhong Wang

**Affiliations:** 1 Jiangxi Province Key Laboratory of Laboratory Medicine, Jiangxi Provincial Clinical Research Center for Laboratory Medicine, Department of Clinical Laboratory, The Second Affiliated Hospital of Nanchang University, Nanchang, Jiangxi China; 2 Queen Mary School, Nanchang University, Nanchang, Jiangxi China; 3 School of Public Health, Nanchang University, Nanchang, Jiangxi China

**Keywords:** Fatty acid metabolism, Tumor microenvironment, Personalized treatment, Prognosis, Acute myeloid leukemia

## Abstract

**Background:**

Acute myeloid leukemia (AML) is the most common malignancy of the hematological system, and there are currently a number of studies regarding abnormal alterations in energy metabolism, but fewer reports related to fatty acid metabolism (FAM) in AML. We therefore analyze the association of FAM and AML tumor development to explore targets for clinical prognosis prediction and identify those with potential therapeutic value.

**Methods:**

The identification of AML patients with different fatty acid metabolism characteristics was based on a consensus clustering algorithm. The CIBERSORT algorithm was used to calculate the proportion of infiltrating immune cells. We used Cox regression analysis and least absolute shrinkage and selection operator (LASSO) regression analysis to construct a signature for predicting the prognosis of AML patients. The Genomics of Drug Sensitivity in Cancer database was used to predict the sensitivity of patient samples in high- and low-risk score groups to different chemotherapy drugs.

**Results:**

The consensus clustering approach identified three molecular subtypes of FAM that exhibited significant differences in genomic features such as immunity, metabolism, and inflammation, as well as patient prognosis. The risk-score model we constructed accurately predicted patient outcomes, with area under the receiver operating characteristic curve values of 0.870, 0.878, and 0.950 at 1, 3, and 5 years, respectively. The validation cohort also confirmed the prognostic evaluation performance of the risk score. In addition, higher risk scores were associated with stronger fatty acid metabolisms, significantly higher expression levels of immune checkpoints, and significantly increased infiltration of immunosuppressive cells. Immune functions, such as inflammation promotion, para-inflammation, and type I/II interferon responses, were also significantly activated. These results demonstrated that immunotherapy targeting immune checkpoints and immunosuppressive cells, such as myeloid-derived suppressor cells (MDSCs) and M2 macrophages, are more suitable for patients with high-risk scores. Finally, the prediction results of chemotherapeutic drugs showed that samples in the high-risk score group had greater treatment sensitivity to four chemotherapy drugs in vitro.

**Conclusions:**

The analysis of the molecular patterns of FAM effectively predicted patient prognosis and revealed various tumor microenvironment (TME) characteristics.

**Supplementary Information:**

The online version contains supplementary material available at 10.1186/s12944-022-01687-x.

## Introduction

Acute myeloid leukemia (AML) is a highly malignant hematological tumor with an unclear pathogenesis and complex genetic mutations that make it highly heterogeneous [1, 2]. AML can be divided into eight French-American-British (FAB) classifications according to its morphological characteristics [3]. Since the end of the twentieth century, deeper and broader basic research, as well as advances in biological techniques, have improved our understanding of AML genetics and pathophysiology, including the analysis of the AML genomic landscape. This revealed different somatic mutation characteristics in AML patients [4, 5]. From 2017 to 2018, the approval of multiple targeted therapies led to milestone achievements for AML therapies [6]. For example, inhibitors of mutant FMS-like tyrosine kinase 3 (FLT3) and isocitrate dehydrogenase 1 and 2 (IDH1 and IDH2) are effective in increasing the response rates and improving the prognosis of patients with AML [7–9]. In addition, induced apoptosis therapy with venetoclax targeting BCL-2 also has achieved significant results [10, 11]. However, as AML progresses, new mechanisms of resistance can appear due to the emergence of subclonals [12, 13]. The activation of some alternative pathways also protects AML cells and promotes drug resistance [14–16], and the TP53 apoptosis network is also involved as a medium to help AML cells resist BCL-2 inhibition [17]. Hence, research and exploration of new targets have important clinical value for the treatment of AML and resistance inhibition.

AML tumor cells are malignant evolutions from bone marrow stem/progenitor cells that can directly affect the blood microenvironment of patients. The tumor microenvironment (TME) of AML is often accompanied by a hypoxic state in which the entry of glucose-derived pyruvate into the tricarboxylic acid (TCA) cycle is inhibited [18, 19]. To adapt to extracellular stimuli, AML cells regulate their own state through metabolic reprogramming, including the generation of acetyl coenzyme A to drive the TCA cycle and oxidative phosphorylation through fatty acid oxidation (FAO), which in turn produces sufficient ATP to meet the needs of growth [20]. The elderly population has the highest incidence of patients with AML [21], and the proportion of adipocytes to stromal cells in the bone marrow microenvironment increases with age, from approximately 20% in young adulthood to approximately 60% by 65 years of age [22]. Studies have shown that AML cells promote their energy metabolism by absorbing fatty acids released from the surrounding adipocytes [23]. Leukemic stem cells (LSCs) greatly increase fatty acid uptake in AML cells by overexpressing the adipose transporter CD36 and by inducing lipolysis in adipocytes to release fatty acids [24]. Thus, fatty acid metabolism (FAM) provides the energy supply for AML cells in anoxic and adipocyte-rich marrow microenvironments.

The targeted inhibition of FAM may shed new light on AML treatments from the perspective of the energy supply. For example, CD36 not only drives FAO to promote the survival of LSCs, but also stimulates LSCs to be enriched in adipose tissue (AT) and to be protected by AT to escape the effects of chemotherapy [24]. Several existing studies have confirmed that sulfo-n-succinimidyl oleate can inhibit fatty acid uptake by cardiomyocytes by binding to CD36 [25, 26], and neutralizing antibodies can block the protein of CD36 to suppress melanoma and breast cancer cell metastasis [27], suggesting that the inhibition of CD36 has potential therapeutic value in the treatment of AML. In several studies, AML cells co-cultured with bone marrow adipocytes were found to highly express the lipid chaperone FABP4 [28]. However, knockdown of FABP4 was able to promote survival in HOXA9/MEIS1-driven leukemia model mice [29]. These findings likewise suggest that FABP4 expression favors AML cell growth. LSCs in AML-relapse patients rely on amino acid metabolism for oxidative phosphorylation, and increased FAO compensates for the absence of amino acid metabolism [30]. These results all suggest that FAM has important biological effects on AML cells. Therefore, a comprehensive understanding of the molecular and TME characteristics of FAM in AML patients can help us to better understand the characteristics of metabolic reprogramming in AML and provide references for clinical decision-making and prognostic evaluation. In this study, we analyze the molecular features of fatty acid metabolism–related genes (FAMGs) and reveal the characteristics of lipid metabolism reprogramming and immune infiltration in different AML patients based on the expression of FAMGs. We also found that the risk-score model constructed by the least absolute shrinkage and selection operator (LASSO) regression accurately predicted the patient prognosis, indicated immune function and clinicopathological differences in different patients, and revealed drugs with potential therapeutic value. Finally, we identified several genes that are strongly associated with cancer development. These results may provide new ideas for the study of metabolic reprogramming and for the treatment of AML.

## Materials and methods

### Data processing

**Fig. 1:**
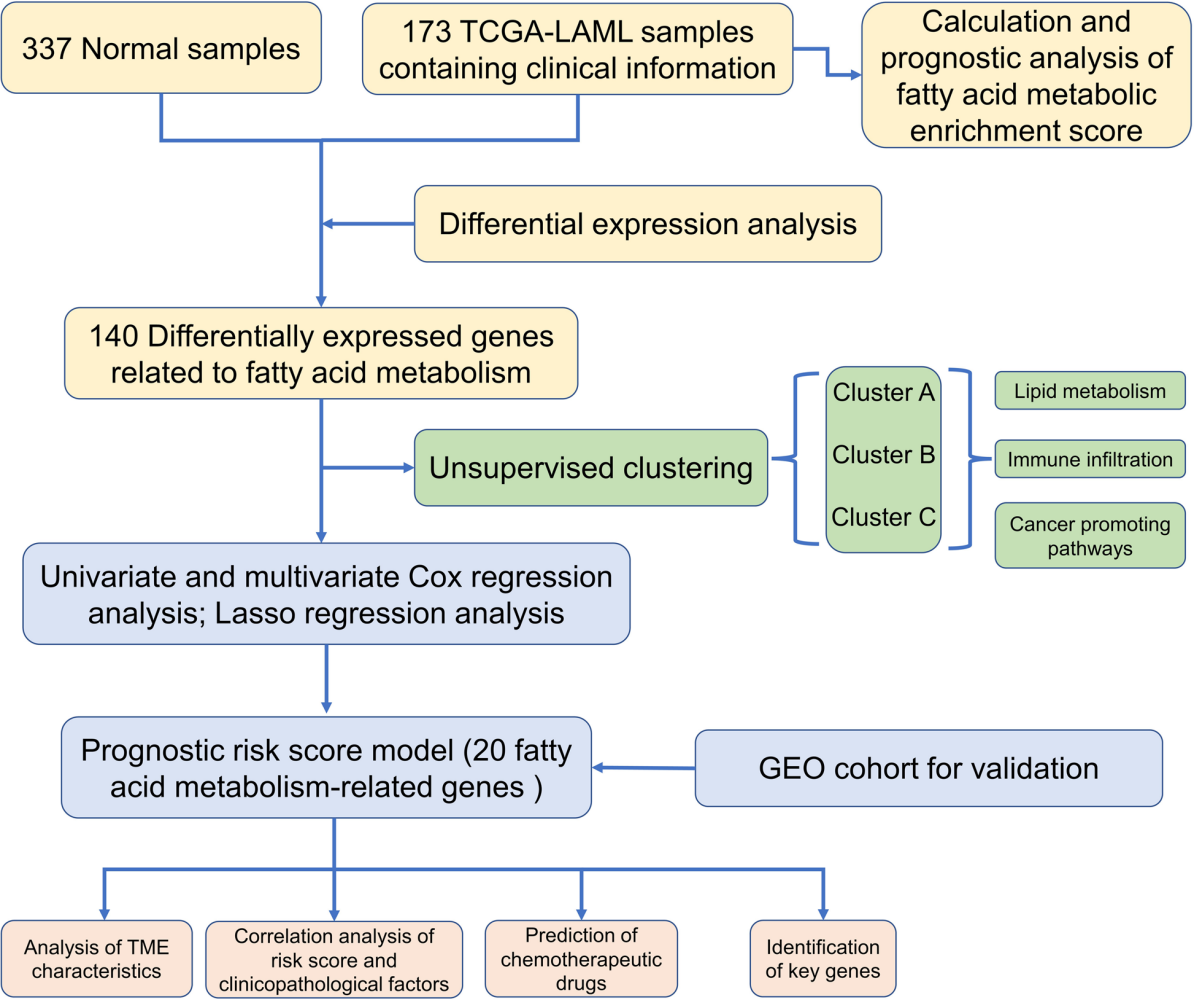
The workflow of this project

The normalized RNA-sequencing data (RSEM tpm) of 173 Acute Myeloid leukemia samples from The Cancer Genome Atlas (TCGA) and 337 whole-blood samples of healthy participants from the Genome Tissue Expression project were downloaded from the University of California Santa Cruz’s XENA database (https://xenabrowser.net/datapages/). The original microarray data "cel" file of 417 AML samples containing clinical information from the GSE12417-GPL96 cohort were downloaded from the Gene Expression Omnibus (GEO) database (https://www.ncbi.nlm.nih.gov/geo/), and we used robust multiarray averaging (RMA) of the "Affy" package to standardize them. For the three GEO datasets (GSE111567, GSE155431, GSE100026), we downloaded the normalized matrix files. Finally, the "HALLMARK_FATTY_ACID_METABOLISM" gene set, which contained 158 FAM-related genes, was downloaded from the MSigDB database (https://www.gsea-msigdb.org/gsea/msigdb/). The workflow of this project is shown in Fig. 1.

#### Gene set variation analysis (GSVA)

GSVA can calculate the enrichment score of a gene set in a single sample according to the overall expression level of genes [31] so as to quantify the activity of a corresponding biological process or signaling pathway. The gene sets of interest, including the immune checkpoints, angiogenesis, nucleotide excision repair, DNA damage repair, mismatch repair, and marker genes of CD8 + effector T-cells, were designed by Mariathasan et al. [32], while the marker genes of myeloid-derived suppressor cells (MDSCs) were previously analyzed by Charoentong et al. [33], and we obtained all of them from the corresponding literature. The gene sets related to lipid metabolism were collected from the Kyoto Encyclopedia of Genes and Genomes (KEGG) database (https://www.kegg.jp/), and the gene sets related to the vascular endothelial growth factor signaling pathway, adhesion, the inflammatory response, hyperoxia, and reactive oxygen species (ROS) were downloaded from the msigdb database (Table S1).

#### Unsupervised clustering of the differentially expressed genes (DEGs) of FAMGs

Based on the expression of 140 DEGs, we clustered AML patients using the consensus clustering algorithm in the "consumusclusterplus" package. This was run 1,000 times to ensure the stability of the results [34]. The algorithm performs hierarchical agglomerative clustering (based on Euclidean distance and Ward's linkage) by analyzing the characterization of gene expression, and identifies patients with similar expression patterns.

#### Calculation of the TME immune cell–infiltration ratio

CIBERSORT, as a deconvolution algorithm, infers the proportion of immune cells in tumor samples through support vector regression based on a set of reference gene-expression values [35]. We used this algorithm to calculate the infiltration level of 22 immune cells including B cells, T cells, natural killer cells, macrophages, DCs, and myeloid subsets in each sample based on the LM22 gene signatures (Table S2).

#### Function analysis and construction of a protein–protein interaction network

A function analysis was performed using the R package “clusterProfiler”. The KEGG enrichment analysis and Gene Ontology (GO) annotation were used to analyze the function of the common DEGs among the clusters and between the high- and low-risk score groups, respectively. A gene set enrichment analysis (GSEA) was used to identify the signaling pathways that differed between the high- and low-risk score groups. The differential genes of the high- and low-risk groups were uploaded to the STRING database (https://string-db.org/) for protein–protein interaction (PPI) network analysis, and the core gene network was then further adjusted using Cytoscape version 3.8.2.

#### Construction of the prognostic risk-score model

We used univariate and multivariate Cox regression analyses to identify the DEGs of the FAMGs significantly related to prognosis for the construction of a risk-score model. Then, LASSO Cox regression analysis was used to remove the redundancy of the prognosis-related genes to prevent overfitting of the model, and a tenfold cross-validation was conducted to determine the penalty parameters (λ) of the model. The following equation was used to calculate the risk score of each sample:

$$Risk\;score= {\sum }_{1}^{i}\left(Coefi*ExpGenei\right)$$

where "Coef" represents the non-0 regression coefficient of each model gene calculated by the LASSO Cox regression analysis, and "ExpGene" is the expression value of the model gene (Table S3).

#### Identification of the DEGs between the clustered subgroups and between the high- and low-risk score groups

Empirical Bayesian methods via the “LIMMA” package were used to analyze the DEGs between the different clustered subgroups or between the high- and low-risk score groups. Genes with adjusted *P* values of < 0.05 and logFC > 1 were considered statistically significantly different.

#### Drug sensitivity prediction

We used the Genomics of Drug Sensitivity in Cancer (https://www.cancerrxgene.org/) database to estimate each patient's sensitivity to chemotherapy drugs [36]. The half-maximal inhibitory concentration (IC_50_) value was used as an index for drug sensitivity assessment using the “pRRophetic” package [37]. The higher the IC50, the less sensitive to the drug.

### Statistical analysis

The Wilcoxon rank-sum test was used to determine the difference between two groups, and the Kruskal–Wallis test was used for multiple groups. Spearman’s method was used for correlation analysis. The "survminer" package divided patients into the high- and low-risk score groups or high- and low-FAMscore groups based on cutoff points for the smallest *P* value. The log-rank test was used to determine the *P* values between the groups in the Kaplan–Meier survival analysis. Univariate and multivariate Cox regression analyses were used to identify the prognostic factors. A receiver operating characteristic (ROC) curve analysis was used to determine the specificity and sensitivity of the related metrics, and the "pROC" package showed the area under the ROC curve (AUC). The "maftools" package was used to characterize the somatic mutations of the AML patients. A two-sided *P* value of < 0.05 was considered statistically significant.

## Results

### Molecular characterization of FAM in the normal and AML samples

**Fig. 2:**
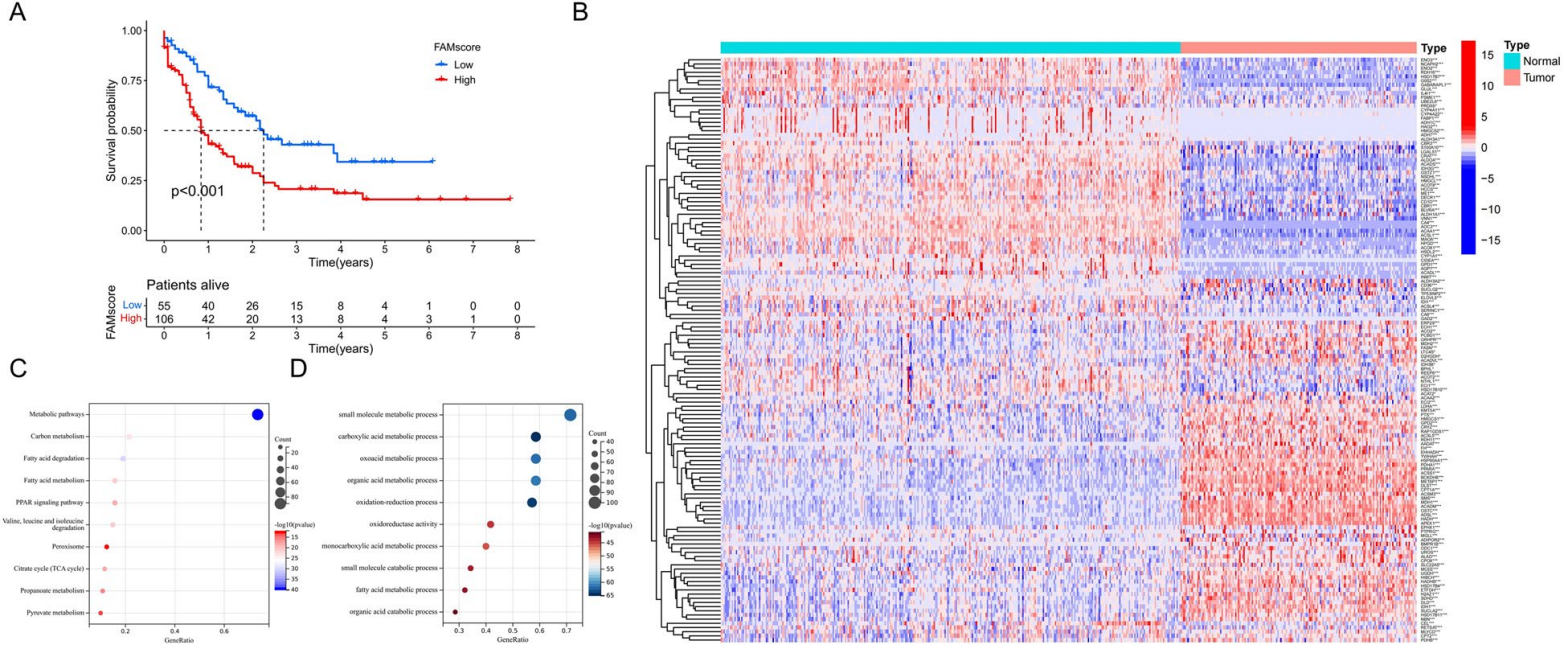
Molecular characterization and prognostic analysis of fatty acid metabolism.** A** Differences in the overall survival (OS) of patients in the high and low FAMscore groups determined using the log-rank test. **B** The difference in the molecular features of the lipid metabolism-related gene (FAMG) expression between acute myeloid leukemia and normal samples determined using the Wilcoxon test. * *P* < 0.05; ** *P* < 0.01; * *P* < 0.001. **C** Kyoto Encyclopedia of Genes and Genomes pathway enrichment analysis of the FAMGs. **D** Gene Ontology annotation of the FAMGs

To analyze the molecular features of FAM in the AML patients, we first calculated an enrichment score for the FAM gene set, named the FAMscore. The survival analysis showed that patients with high FAMscore values had a significantly worse prognosis (Cox regression analysis; univariate: HR = 4.33 (1.85–10.14), *P* = 0.00071; multivariate: HR = 6.00 (2.14–16.86), *P* = 0.00066) (Fig. 2A), indicating that FAM was progressively enhanced with tumor progression in AML patients. Up-regulation of FAM was considered an important cause of Venetoclax with azacitidine (ven/aza) resistance in AML patients [38]. We examined the RNA-seq data from nine AML patients treated with ven/aza [38], and three progressors had significantly higher FAMscores than six responders (Figure S1A). In addition, as the disease progresses in patients with chronic myeloid leukemia (CML), CML cells rapidly proliferate by enhancing fatty acid metabolism. We analyzed the differences in the FAMsocres among patients of different stages in our own CML cohort data [39]. The results showed that the CML patients had significantly higher FAMscores than healthy people, and the FAMscores of those in the blast crisis (BC) phase were higher than those in the chronic phase (CP) (Figure S1B). In another dataset containing plasma, the fatty acid content of healthy individuals, and the RNA-seq data of peripheral blood mononuclear cells (PBMCs) [40], we observed no difference in the FAMscores of the PBMCs among the groups with different n-3 polyunsaturated fatty acid (PUFAs) contents. Compared with the low/high n-6 PUFAs content group or the low/high saturated fatty acid (SFA)/PUFA ratio group, the FAMscore of the middle group was higher (Figure S1C). We further analyzed the relationship between FAMscore and clinicopathological factors. The results showed that among all the FAB subtypes, M5 had the highest FAMscore, and that patients with high white blood cell (WBC) counts and high proportions of peripheral blood (PB) blasts also had significantly higher FAMscores (Figure S2A). In addition, patients with FLT3 mutations had higher FAMscores than wild-type patients (Figure S2B).

The expressions of 71 FAMGs were upregulated and those of 69 were downregulated in the AML samples compared with the normal control samples (Fig. 2B). KEGG analysis showed that these DEGs were primarily enriched in the metabolic pathways, FAM and fatty acid degradation, amino acid breakdown, and the TCA cycle (Fig. 2C). The Gene Ontology (GO) annotations also focused on a large number of biological processes, such as FAM, small-molecule metabolism, and the redox of organic acids (Fig. 2D). These results revealed aberrant alterations in the metabolic genomics in the AML cells, which may be involved in the development of AML.

### Differences in the biological characteristics between the molecular subtypes based on FAMG clustering

**Fig. 3:**
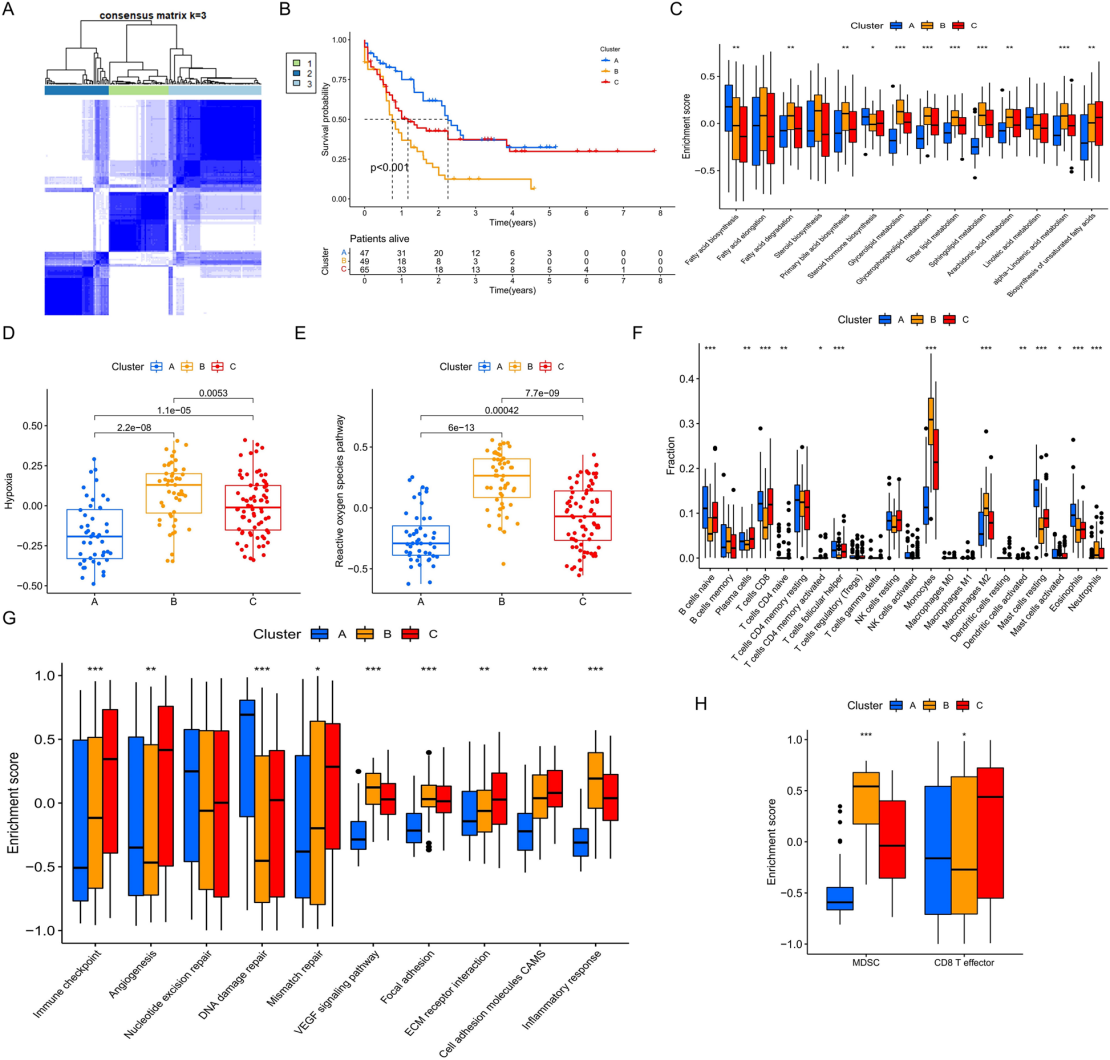
Identification of fatty acid metabolism (FAM)-related molecular subtypes. **A** Consensus matrices for k = 3. **B** Survival analysis of the different FAM-related molecular subtypes performed using the log-rank test. **C**–**H** The enrichment score of the signaling pathway or infiltration level of the tumor microenvironment cells in the different FAM-related molecular subtypes; **C**: lipid metabolism pathways, **D**: hypoxia pathway, **E**: reactive oxygen species pathway, **F**: 22 tumor microenvironment cells, **G**: other cancer–promoting pathways, and **H**: myeloid-derived suppressor cells and CD8 + effector T-cells. Kruskal–Wallis test, * *P* < 0.05; ** *P* < 0.01; * *P* < 0.001

To better understand the FAM profile of the AML patients, we performed a consensus clustering of the AML patients in the TCGA cohort based on the expression of 140 DEGs. The clustering results showed that the 173 AML patients could be divided into three clusters, and that this clustering had the greatest cluster stability (Fig. 3A). Survival analysis showed that the patients of cluster B had the worst prognoses and the highest FAMscore values (Fig. 3B). To better explore the lipid metabolism characteristics of patients with different molecular subtypes, we collected the gene sets of 14 lipid metabolism pathways and calculated the enrichment score of each patient (Fig. 3C). We observed that the biosynthesis of fatty acids and steroid hormones in cluster A were increased, while the metabolism of multiple lipids, including fatty acids, glycerides, glycerophospholipids, ether lipids, and sphingolipids, was significantly more active in cluster B than the other two clusters. However, in the metabolic pathways associated with unsaturated fatty acids, we observed that arachidonic acid and α-linolenic acid were metabolically enhanced in cluster B, linoleic acid was metabolically most active in cluster A, and unsaturated fatty acid biosynthesis was increased in cluster C. These findings revealed the heterogeneity of lipid metabolism in AML patients. In addition, the TME is often accompanied by hypoxia, which in turn induces mitochondria to produce a large amount of ROS and causes oxidative stress. We used the same approach and found higher hypoxia levels and more ROS generation in cluster B, which may correlate with the increased lipid metabolism in cluster B (Fig. 3D and E).

We further analyzed the immune-infiltration characteristics of the different molecular subtypes (Fig. 3F). The best prognosis for patients in cluster A could be related to the enrichment of a large number of adaptive immune cells—such as naïve B-cells, CD8 + T-cells, and follicular helper T-cells—and innate immune cells—including NK cells, mast cells, and eosinophils. A similar proportion of immune cells, albeit slightly less infiltrated, was also found in cluster C and cluster A, and the prognostic status of a patient in cluster C was slightly worse as a result. In addition, in cluster B, a high proportion of monocytes, M2 macrophages, and neutrophils exhibited strong inflammatory signals. Importantly, the development of inflammation in the TME tends to suppress immune function as well as generate drug resistance and manifest a poor prognosis, suggesting a potential mechanism that may be mediating the inferior survival seen for patients in cluster B.

Finally, we analyzed other signaling pathways that may be associated with AML tumor development, including immune checkpoints, angiogenesis, DNA damage repair, cell adhesion, and inflammatory responses. Through an enrichment analysis of these pathways, we found that cluster C overexpressed the immunological checkpoint, activated the tumor angiogenesis signal, and enhanced cell adhesion (Fig. 3G). Cell infiltration analysis by GSVA showed that cluster B contained more MDSCs, while CD8 + effector T-cells were significantly enriched in cluster C (Fig. 3H). These signatures all demonstrated that when AML cells express more immunosuppressive and cell adhesion molecules at the genomic level, they may be more prone to immune escape or adherence to a safe living environment, and this phenomenon may explain why the massive immune cell infiltration in cluster C was associated with a worse prognosis relative to cluster A. The high activity of the inflammatory response pathway in cluster B also corresponded to the enrichment of inflammatory immune cells, and the pro-inflammatory effect of MDSCs can also further produce immunosuppression.

### The risk-score model is robust for prognosis prediction

**Fig. 4:**
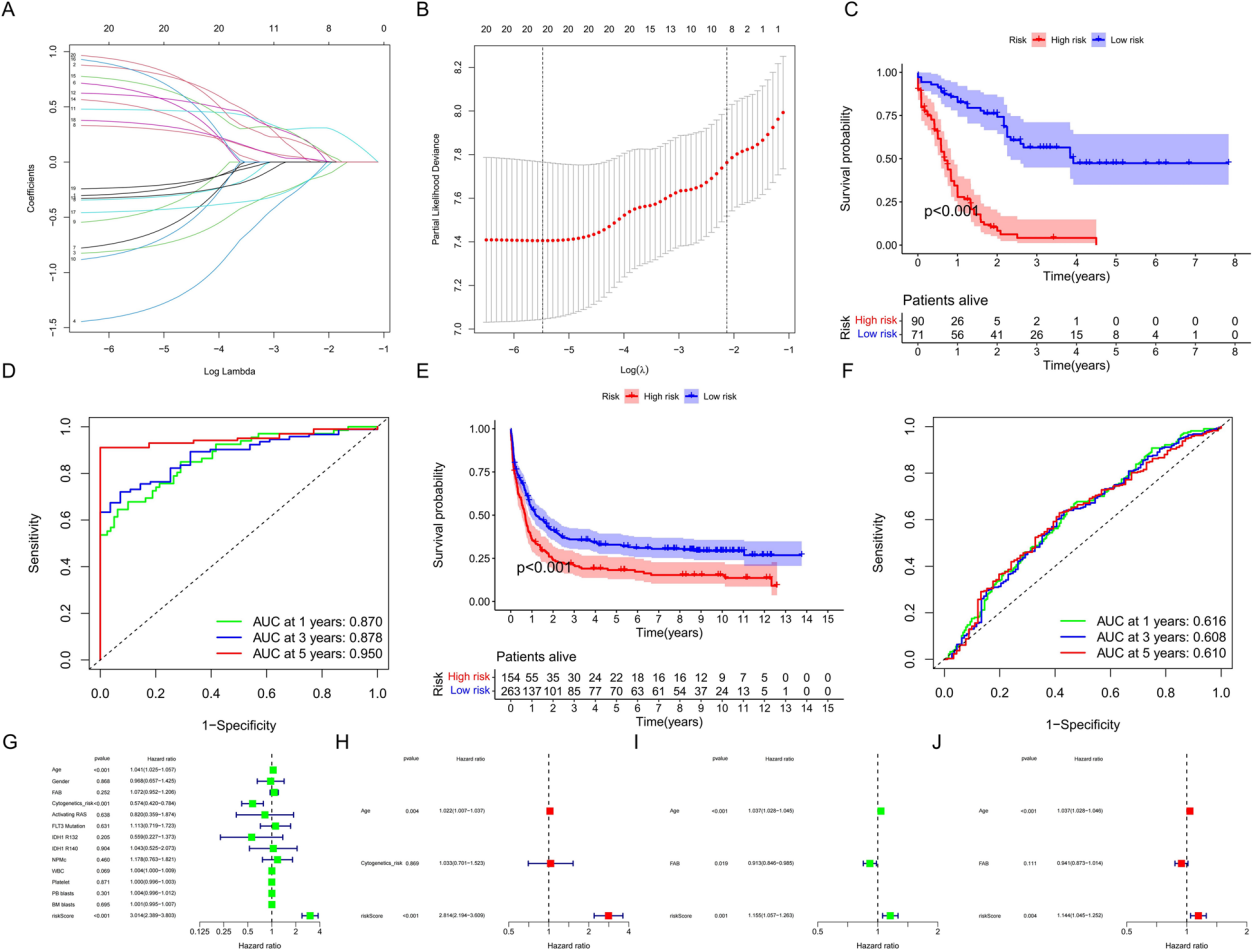
Construction and validation of the risk-score model. **A** Determination of the log(λ) corresponding to the minimum tenfold cross-validation error point. **B** The non-0 coefficient corresponding to the same log(λ) value. **C** Survival analysis between the high-risk and low-risk score groups in The Cancer Genome Atlas (TCGA) cohort performed using the log-rank test. **D** Time-dependent receiver operating characteristic (ROC) curve analysis of the risk score in the TCGA cohort. **E** Survival analysis between the high-risk and low-risk score groups in the Gene Expression Omnibus (GEO) cohort performed using the log-rank test. **F** Time-dependent ROC curve analysis of the risk score in the GEO cohort. **G**, **I** Univariate independent prognostic analyses of the clinicopathologic factors and risk score; G: TCGA cohort and I: GEO cohort. **H**, **J** Multivariate independent prognostic analyses of the clinicopathologic factors and risk score; **H**: TCGA cohort, and J: GEO cohort

To better predict the prognosis and characterize the TME, we constructed a prognostic risk-score model to assess the individual status of patients. In the TCGA cohort, univariate and multivariate Cox regression analyses identified a gene significantly associated with the prognosis of AML patients (Figure S3A and S3B), and LASSO regression analysis further reduced dimensionality and screened out 20 FAMGs for model construction (Fig. 4A and B). We calculated the risk score of each patient using the model equation. The "survminer" package was used to calculate the cutoff value when the *P* value was the smallest, and we divided the 161 patients with survival information into high-risk score and low-risk score groups. As the risk score increased, the survival time shortened and the number of deaths increased (Figures S4A and B). The survival analysis showed that patients with high risk scores had significantly worse prognoses (Fig. 4C). A heatmap showed the expression levels of the model genes in the high and low risk score groups (Figure S4C). The time-dependent ROC curve analysis revealed that the AUC values ​​at 1, 3, and 5 years were 0.870, 0.878, and 0.950, respectively, indicating that the model had a high prognostic prediction accuracy (Fig. 4D). In another Gene Expression Omnibus (GEO) validation cohort, GSE12417-GPL96, we also observed a significantly worse prognosis for patients in the high-risk group (Fig. 4E) while the number of deaths and the expression of model genes exhibited the same risk-score distribution as the TCGA cohort (Figure S4D–S4F), with AUC values ​​for 1, 3, and 5 years of 0.616, 0.608, and 0.610, respectively (Fig. 4F). The univariate and multivariate independent prognostic analyses further indicated that risk score could serve as an independent predictor for patient prognosis (Fig. 4G–J). In conclusion, with further validation in the GEO cohort, our constructed risk-score model demonstrated a stable prognostic predictive value.

### Correlation analysis of the risk score and clinicopathological factors

**Fig. 5:**
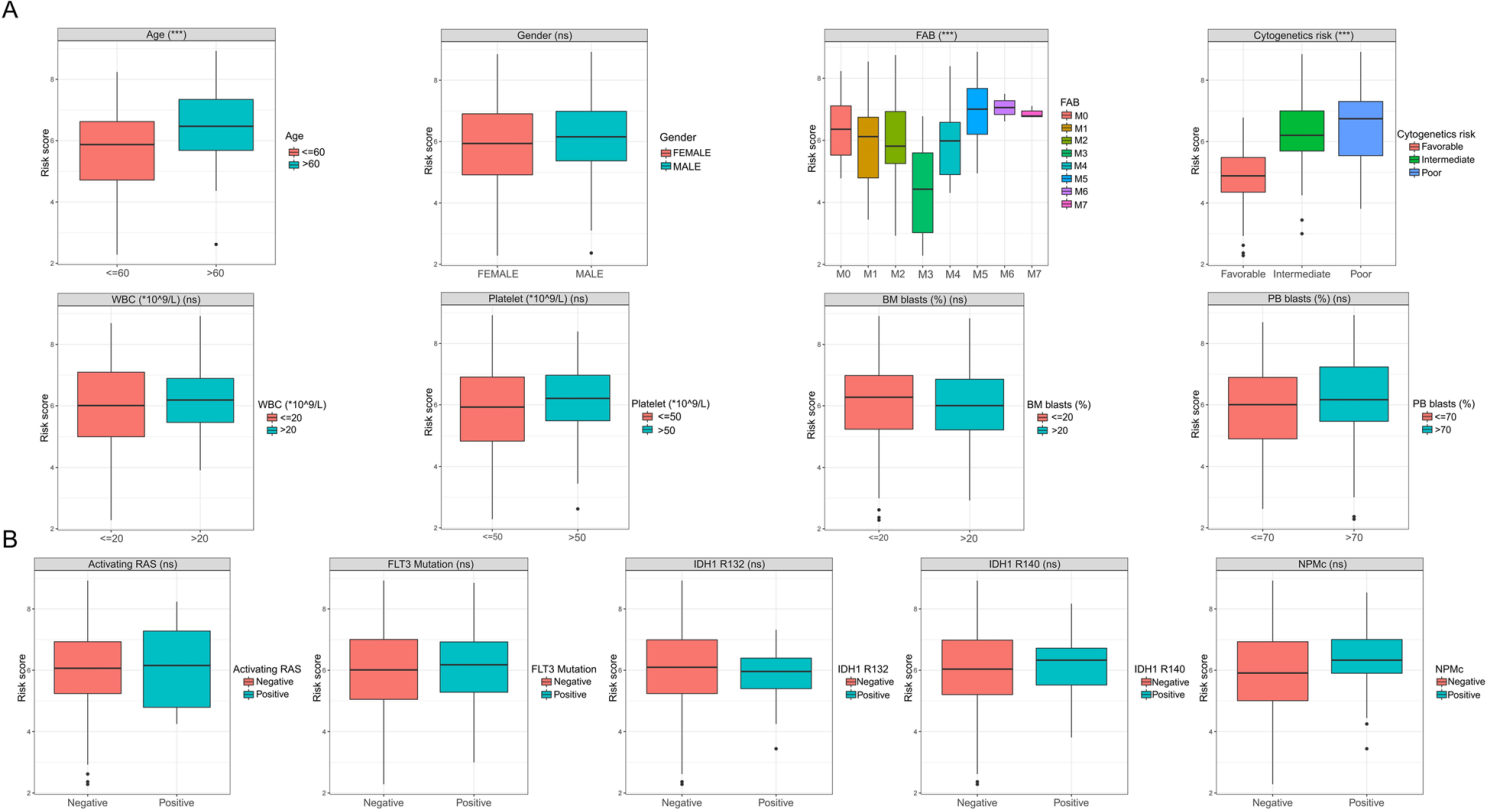
Differences in the risk scores among patients with different clinicopathological characteristics. **A** Risk-score differences in different individual characteristics such as age, gender, French-American-British classification, cytogenetic risk, white blood cell or platelet count, and bone marrow or peripheral blood blast count. **B** Risk-score differences in the different somatic variation signatures (e.g., RAS-activating, FLT3/IDH mutation, or cytoplasmic nucleophosmin profile). * *P* < 0.05; ** *P* < 0.01; * *P* < 0.001

We further compared the relationships of the different clinicopathological factors with the risk scores. Among some individual characteristics (Fig. 5A), we found no significant differences in the risk score between the different genders or between patients with different peripheral white blood cell and platelet counts or bone marrow and peripheral blood blast counts. However, patients of advanced age (≥ 60 years old) had significantly higher risk scores than those aged < 60 years old. In the FAB classification, we observed that the risk score decreased sequentially from M0 to M3 and increased sequentially from M3 to M7. In addition, the worse the cytogenetic risk, the higher the risk score. Among the different AML-related gene-mutation signatures (Fig. 5B), we observed no difference in risk scores between the mutation-positive patients—such as Ras-activating, FLT3-mutated, IDH1-mutated, and nucleophosmin cytoplasmic (NPMc) profiles—compared with the mutation-negative patients.

### The risk score indicated immune infiltration and can guide clinical treatment

**Fig. 6:**
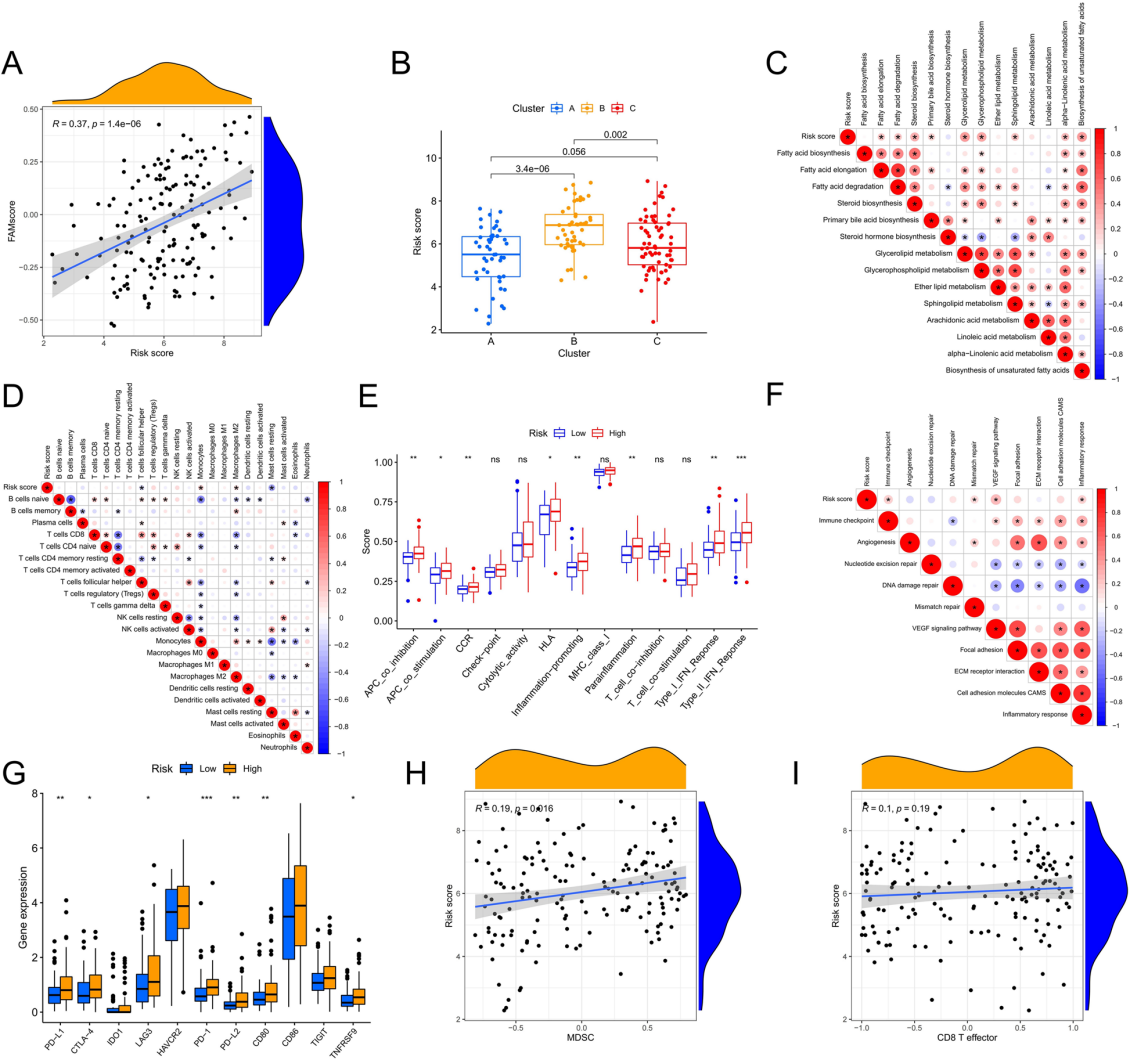
Correlation analysis between the tumor microenvironment characteristics and the risk score. **A** Correlation analysis between the FAMscore and the risk score. **B** Differences in the risk scores of the clustering subtypes. **C** Correlation analysis between lipid metabolism and the risk score. **D** Correlation analysis between the proportion of immune cell infiltration and the risk score. **E** Differences in the risk scores between the different immune functions. **F** Correlation analysis between other cancer-promoting signaling pathways and the risk score. **G** Differences in the risk scores of different immune checkpoints. **H** Correlation analysis between the infiltration level of myeloid-derived suppressor cells and the risk score. **I** Correlation analysis between the infiltration level of CD8 + effector T-cells and the risk score. * *P* < 0.05; ** *P* < 0.01; * *P* < 0.001

High-risk scores are strongly associated with a poor prognosis, and we further analyzed the immune and other features of the TME in patients with different risk scores in an attempt to mine therapeutic targets for different patients. The risk score was significantly and positively correlated with the FAMscore (Fig. 6A), indicating that a higher risk score correlated with stronger FAM activity. Among the three clustering subgroups, patients in cluster B had the highest risk scores, performed well in accordance with the distribution characteristics of the FAMscore, and the risk score also discriminated patients in different subtypes well (Fig. 6B). The risk score was positively associated with aspects of lipid metabolism such as fatty acid elongation, degradation, glyceride metabolism, and the synthesis of unsaturated fatty acids (Fig. 6C). Among the immune cell–infiltration signatures, higher risk scores were associated with less infiltration of follicular helper T-cells and resting mast cells and more infiltration of monocytes and M2 macrophages (Fig. 6D). Among the immune function–related pathways, antigen presenting cell (APC) co-inhibition, APC co-stimulation, C–C motif chemokine receptor (CCR) and human leukocyte antigen (HLA) activity, inflammation promotion, para-inflammation, the type I interferon (IFN) response, and the type II IFN response were more active in the high-risk score group (Fig. 6E and Table S 4). We then compared the activity of the other signaling pathways that promote tumor development with the risk score, and we found that the activity of immune checkpoints, mismatch repair, vascular endothelial growth factor signaling pathways, and the inflammatory response increased in correlation with a higher risk score (Fig. 6F). These results demonstrated a state of vigorous lipid metabolism, immunosuppression, and the development of inflammation in the TME of patients with high-risk scores. To better guide the decision-making of clinical immunotherapy, we further analyzed the expression levels of immune checkpoints; notably *PD-L1*, *CTLA-4*, *IDO1*, *LAG3*, *HAVCR2*, *PD-1*, *PD-L2*, *CD80*, *CD86*, *TIGIT*, and *TNFRSF90* that have been mentioned as immune checkpoint–related genes in relevant studies [32]. The expressions of *PD-L1*, *CTLA-4*, *LAG3*, *PD-1*, *PD-L2*, *CD80*, and *TNFRSF90* were significantly upregulated in the high-risk score group (Fig. 6G). In addition, high-risk scores were accompanied by the infiltration of more MDSCs (Fig. 6H), but a change in effector T-cells was not obvious (Fig. 6I). Based on this, we suggest that patients with high-risk scores may benefit from certain treatment modalities, including FAM inhibition, immune checkpoint therapy, and targeted inhibition of M2 macrophages and MDSCs.

**Fig. 7:**
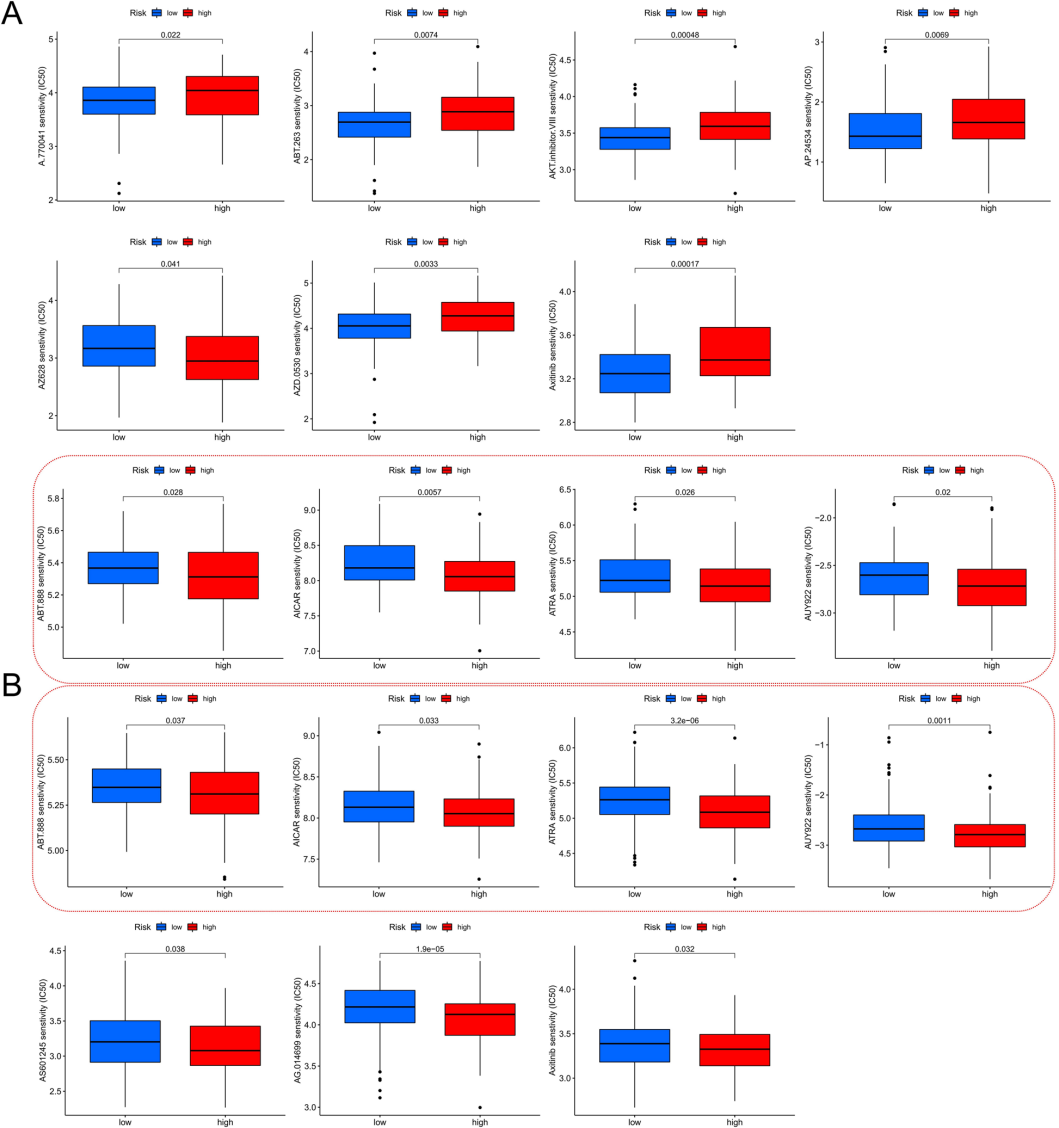
Prediction of drugs for the high-risk and low-risk score groups. **A**, **B** Sensitivity analysis of anti-cancer drugs in the high and low FSMscore groups performed using the Wilcoxon test; **A**: The Cancer Genome Atlas cohort and **B**: The Gene Expression Omnibus cohort. In the red dashed box are drugs with the same sensitivity differences predicted by both the TCGA and GEO cohorts

In addition, we evaluated the difference in the IC_50_ values of 138 chemotherapeutic agents in the high- and low-risk score groups. We simultaneously compared the sensitivity to chemotherapeutic agents between the high- and low-risk groups in the TCGA and GEO cohorts. The data revealed a significant sensitivity difference between the TCGA and GEO cohorts (11 and seven chemotherapeutic agents, respectively), with both cohorts identifying four chemotherapeutic agents with greater sensitivity in the high-risk score group, including ABT.888 (veliparib, a polyADP-ribose] polymerase inhibitor), 5-aminoimidazole-4-carboxamide ribonucleoside (AICAR, an adenosine monophosphate kinase activator), all-trans-retinoic acid (ATRA), and AUY922 (luminespib, an HSP90 inhibitor) (Fig. 7A and B).

### Identification of key genes with significant differences between the high- and low-risk score groups

**Fig. 8:**
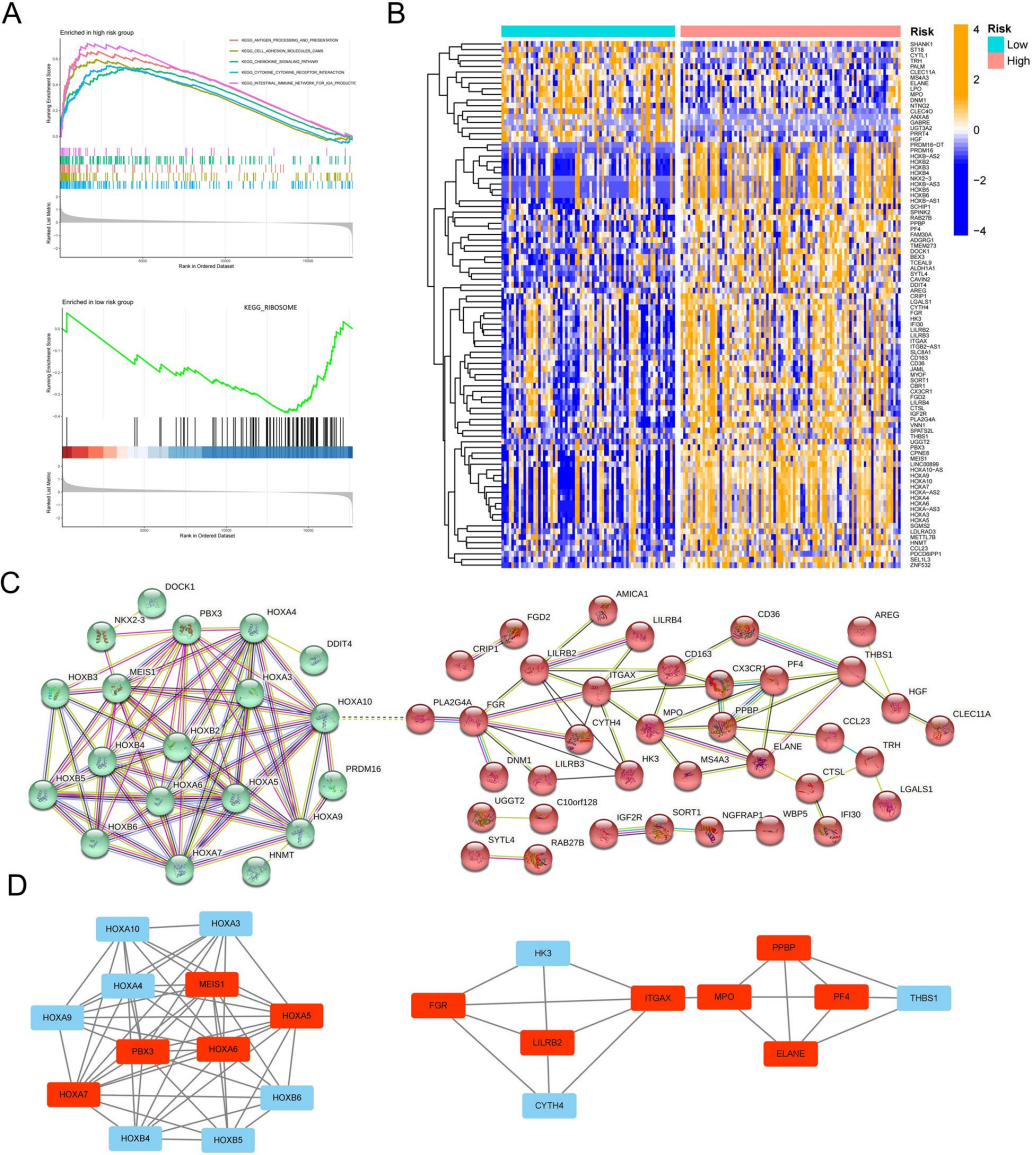
Identification of the significant difference pathways and key genes between the high- and low-risk score groups. **A** The gene set enrichment analysis revealed pathways with significant enrichment differences between the high- and low-risk score groups. **B** The heatmap showed the expression of genes with significant differences between the high- and low-risk score groups. **C** A protein–protein interaction network showed the interaction and subnetworks of the differentially expressed genes. **D** Identification of the core genes with the highest connectivity in the subnetwork. The green subnetwork is on the left, and the red subnetwork is on the right

To better reveal genes with obvious expression differences in the high- and low-risk score groups and identify potential key genes involved in AML tumor development, a GSEA was conducted. Its results showed that the top five signaling pathways with the smallest enrichment-difference *P* values in the high-risk score group were as follows: cytokine receptor interaction, cell-adhesion molecules, antigen processing and presentation, the chemokine signaling pathway, and the intestinal immune network for immunoglobulin A production. In the low-risk score group, only one ribosome-related signaling pathway was enriched (Fig. 8A). These results again suggested that tumor development in AML is accompanied by significant inflammatory and immunobiological process changes.

We further identified 94 DEGs in the high- and low-risk score groups (Fig. 8B) and constructed a PPI network. We then used k-means clustering to further divide the network into two subnetworks (Fig. 8C and Table S5). In the green subnetwork, we were surprised to find a large enrichment of the homeobox (*HOX*) family genes that have been extensively studied to prove that high expression of these genes promotes leukemia development. The KEGG enrichment analysis showed that the green-subnetwork genes were primarily involved in transcriptional dysregulation, metabolic pathways, cell-adhesion molecule signaling pathways, and the mammalian target of rapamycin and PI3K Akt signaling pathways in cancer (Figure S5A). The GO annotations were primarily enriched in the regulation of the RNA biosynthetic process, definitive hemopoiesis, and immune system development (Figure S5B). In the red subnetwork, the results of the KEGG analysis primarily enriched viral protein interactions with cytokine and cytokine receptors, the chemokine signaling pathway, the B-cell receptor signaling pathway, cytokine-cytokine receptor interactions, and ECM receptor interactions (Figure S5C). GO annotations were primarily found in entries such as vesicle-mediated transport, neuronal activation induced in immune responses, myeloid leukocyte activation, and immune responses (Figure S5D). By combining these two subnetworks’ enriched pathways and biological processes, abnormal transcription regulation, abnormal cytokine expression, increased adhesion, metabolic reprogramming, and increased vesicle transport were deemed to be characteristic signatures for the development of AML tumors. In addition, we identified the genes with the most connections as key genes in the subnetwork (Fig. 8D). In the green subnetwork, *HOXA5*, *HOXA6*, *HOXA7*, *PBX3*, and *MEIS1* were located at the core of the network, while in the red subnetwork, *FGR*, *LILRB2*, *ITGAX*, *MPO*, *PPBP*, *PF4*, and *ElANE* had the highest connectivity.

## Discussion

Lipid metabolism, especially FAM, is an important process in cell life activities. After obtaining nutrients, cells will process and convert them into intermediates of various metabolic pathways, and these intermediates play a role in cell membrane synthesis, energy reserve, and the production of active molecules [41]. As an important form of the metabolic reprogramming of tumor cells, abnormal changes in FAM will affect the reactivity and activity of other metabolic pathways at the same time and have a great impact on the bioenergetics, proliferation, growth, and signal transduction of tumor cells [18]. In addition, lipid metabolism also affects the migration and invasion of tumor cells in the TME, induces tumor angiogenesis, promotes tumor cells to evade the surveillance of body immunity, and increases drug resistance [42]. In AML, the anoxic bone marrow microenvironment inhibits the ratio of acetyl coenzyme A after glycolysis and thus limiting the tricarboxylic acid (TCA) cycle. However, the activation of FAO will promote more ATP production, which is beneficial to the growth and survival of leukemia cells [18]. Currently, most studies on FAM and the occurrence and development of AML have focused on a single molecule, and there is a lack of systematic evaluation of the relationship between the FAM-related gene set and the pathological characteristics of AML. Therefore, exploring the molecular model of FAM and its relationship with biological processes, such as immunity and inflammation, may lead to a better understanding of the impact of FAM on the development of AML.

Our study innovatively integrated FAMGs and revealed the pathobiological states of metabolic reprogramming, immune escape, and inflammation development in the TME of different AML patients based on the transcriptome expression levels of these genes, representing the first attempted exploration in AML research. We first found that the FAMscore was associated with a poor prognosis in AML patients, suggesting that the exacerbation of AML may be accompanied by increased FAM. Ven/aza-resistant AML cells exhibited highly active fatty acid metabolism, consistent with our calculated high FAMscore. In our CML cohort, a high FAMscore also reflected enhanced fatty acid metabolism and the rapid proliferation of malignant phenotypes of CML cells. This is because CML cells require a lot of energy and promote the fluidity of cell membranes by increasing the content of unsaturated fatty acids [43]. All these indicate that the FAMscore has certain accuracy in predicting the fatty acid metabolism activity of leukemia cells. We further assessed the correlation between the plasma fatty acid content and the FAMscore in PBMCs in healthy individuals, and the relationship between the FAMscore and different levels of n-6 PUFAs, or of that between different ratios of SFA/PUFA, was “bell shaped”, as appears to be the case for the relationship between the status of many nutrients and the PBMC function [44], the excessive intake of certain fatty acids may inhibit the function of PBMC. However, there are still few studies and data regarding the relationship between lipids in the plasma and the PBMC function. This correlation analysis may have reference value for revealing the relationship between fatty acids in peripheral blood and the metabolism of AML cells. In the correlation analysis between FAMscore and clinicopathological factors, M5 AML, which originated from the malignant transformation of primitive monocytes, had the highest FAMscore, which may be related to the energy supply required for its inflammatory effect [45]. And these AML cells can fully utilize fatty acid oxidation to activate cancer-promoting signaling pathways such as AMPK, and up-regulate genes such as *PPARγ, FABP4, CD36* and *BCL2* genes to promote their own survival and proliferation [28]. FLT3-mutated AML patients also exhibited high FAMscore. One study showed that L-carnitine was significantly more abundant in AML cells from FLT3 mutant patients compared to wild-type patients, and that it produces cellular energy through fatty acid beta-oxidation [46]. Similar features were observed in patients with non-small cell lung cancer [47].

We next conducted a cluster analysis based on the FAMGs present with differential expressions in AML patients and normal individuals. We found that patients with different levels of FAM had different TME features; more specifically, patients with reduced FAM had abundant immune cell infiltration, lower activities of various lipid metabolism pathways, and possessed the best prognostic status, corresponding to cluster A. With further enhancement of FAM in cluster C, patient prognosis became poorer and patients began to exhibit immunosuppression, including highly expressed immune checkpoints, the strongest cell-adhesion ability of all the clusters, and progressive inflammation, along with the biosynthesis of unsaturated fatty acids, which are the biological behaviors that prompt AML cells to evade immune cell attack. This trend may also be responsible for the massive infiltration of CD8 + effector T-cells in the TME of such patients through feedback regulation. Patients in cluster B had the strongest FAM of all the clusters, along with significantly increased activity of other lipid metabolism pathways; this was indicated by less tumor killer immune-cell infiltration in the TME of these patients, along with increased infiltration of M2 macrophages promoting the development of inflammation, as well as massive monocyte infiltration. This suggested a highly developed inflammatory condition, which was also confirmed by the hyper-responsive status of the inflammatory signaling pathways. The malignant development of inflammation may be an important reason for the suppression of immune cell responses, and the abundant infiltration of MDSCs also suggests a high degree of immunosuppression in such patients [48], who therefore also exhibit the worst prognosis. The status of the development of immunosuppression and inflammation in the TME of patients can be further assessed by monitoring the levels of FAM in AML cells.

To better link FAM to the prognosis and TME characteristics of AML patients, we developed a risk-score model based on FAM-related genes. This risk score was highly positively correlated with FAM and could accurately predict the prognosis of patients; this was verified in another AML cohort. We focused on the association of the risk score with the TME characteristics, with a higher risk score being associated with a stronger metabolism of fatty acids and glycerides, glycerophospholipids, and a greater biosynthesis of unsaturated fatty acids. In addition, patients with high risk scores had significantly higher expression levels of immune checkpoint-related genes, such as *PD-1*, *PD-L1*, *PD-L2*, and *CTLA-4*, and significantly increased infiltration of immunosuppressive cells, such as MDSCs and M2 macrophages. Immune functions, such as inflammation promotions, para-inflammation, and type I/II IFN responses, were also significantly activated in the high-risk score group, suggesting that AML cells in this group can effectively receive immune stimulation. Therefore, immunotherapy targeting immune checkpoints and immunosuppressive cells is of significance as a reference for the clinical treatment of patients with high-risk scores. We similarly observed greater treatment sensitivity in patients with high-risk scores to four chemotherapeutic agents, with ABT-888 (veliparib) targeting poly (ADP-ribose) polymerase exhibiting intermediate cytotoxic activity in the AML cells and being capable of enhancing the growth inhibitory effect of the alkylating agent temozolomide on AML primary leukemia cells [49, 50]. All-trans-retinoic acid (ATRA) predominantly promotes oncoprotein PML-RARα knockdown to eliminate AML M3 leukemia cells [51], and this can significantly improve the prognosis of M3 patients. 5-aminoimidazole-4-carboxamide ribonucleoside (AICAR), as an agonist of adenosine monophosphate–activated kinase, can promote the differentiation and inhibit the proliferation of leukemia cells, and its combination with ATRA can promote the differentiation of various classifications of AML cells [52, 53]. In the form of molecular chaperones, heat shock protein 90 (Hsp90) synergizes with the signaling pathways involved in cancer cell proliferation, growth, and cellular adaptation, and the Hsp90 inhibitor AUY922 (luminespib) significantly promotes the degradation of the KG-1a fusion oncoprotein FOP2-FGFR1 and inhibits the PI3K and IKK signaling pathways in AML cell lines. The combination of AUY922 with cytarabine also significantly improves outcomes in AML [54, 55]. These studies all confirmed the therapeutic value of four chemotherapeutic agents in AML, and the somatic mutational signatures or classification characteristics of patients with high-risk scores, used alone or in combination, have implications for clinical decision-making.

The TME differed significantly between patients in the high- and low-risk score groups, and we further explored the genes influencing these biological changes. We observed that most members of the *HOX* gene family had consistently high expression in the high-risk group, and many studies have confirmed that the high expression of *HOX* family genes blocks hematopoietic cell differentiation as an important influence on AML tumor development [56–58]. Mutant *NPM1* maintains the leukemic state through the expression of *HOX *[59, 60], while mixed-lineage leukemia-specific chromosomal aberrations or abnormalities interfere with normal hematopoiesis by regulating the overexpression of *HOX* genes [61]. In addition, the PBX proteins and MEIS proteins can regulate the transcription of downstream target genes by binding to HOX proteins to form dimers or trimers [62, 63]. We observed that the *PBX3* gene and the *MEIS3* gene were highly expressed in the high-risk score group. We also observed the aberrant overexpression of *MEIS1* and *HOXA9*, members of the same family as *MEIS3*, that is highly effective in transforming hematopoietic progenitors and driving mice toward a lethal leukemia [64, 65]. In addition, their overexpression is also required for the maintenance and induction of mixed-lineage leukemia [66]. However, the role of *MEIS3* in the development and progression of AML has been less studied. To date, *PBX3* has been confirmed to promote leukemogenesis as a cofactor of HOXA9 [67]. However, the selective inhibition of other genes, such as the Src family kinase *FGR*, can hinder AML cell growth [68], and genes such as integrin *ITGAX* and *PPBP* have been less frequently reported in AML. Furthermore, these genes all showed high transcriptome levels of expression in the high-risk score group. In conclusion, the differential genes between the high- and low-risk score groups are widely reported to be closely related to the occurrence and development of AML tumors, and an integrative analysis and combined targeted inhibition of the genes at the core of these networks may have an instructive role in the study of AML mechanisms and treatments.

In summary, based on the expression of FAMGs, this project revealed the molecular characteristics of the TME biological signals in different AML patients, such as FAM, immune infiltration, and inflammation. The risk-score model can accurately assess the prognosis of patients and indicate tumor-related pathological features. Higher risk scores may reflect stronger lipid metabolism, immunosuppression, and the development of inflammation. The high expression of immune checkpoints in the high-risk score group suggests that these patients may be more sensitive to immunotherapy, and the sensitivity prediction of multiple chemotherapeutic drugs can also provide reference for further research. These findings not only provide a high-precision prognostic assessment model for the clinic, but also provide a new perspective for the study of metabolic reprogramming in AML. However, there are still many limitations in this study. The conclusions of these analyses are based on public datasets. We need to verify in more clinical or independent cohorts, and further confirm through in vivo and in vitro experiments. In the future, we will further explore the effect of inhibiting FAM on AML cells and the mechanisms associated with leukemia stem cell-induced relapse.

## Conclusions

This project revealed significant differences in the TME characteristics among AML patients with different FAM patterns. Moreover, the risk-score model we constructed effectively predicted the prognosis of AML patients, and indicated the activity of FAM and the characteristics of immune infiltration in the TME. And from a genomics perspective, samples in AML patients with high-risk scores were predicted to be adaptive to immunotherapy and high sensitivity to four chemotherapeutic agents.

## Supplementary Information

**Additional file 1.**

**Additional file 2.**

## Data Availability

All data used in this work can be acquired from the Gene-Expression Omnibus (GEO; https://www.ncbi.nlm.nih.gov/geo/) and the UCSC XENA database (https://xenabrowser.net/datapages/).

## Abbreviations

AML: Acute myeloid leukemia
TME: Tumor microenvironment
MDSCs: Myeloid-derived suppressor cells
FAM: Fatty acid metabolism
TCGA: The Cancer Genome Atlas
GETx: Genome Tissue Expression
GEO: Gene Expression Omnibus
GO: Gene Ontology
GSEA: Gene set enrichment analysis
DEGs: Differentially expressed genes
KEGG: Kyoto Encyclopedia of Genes and Genomes
IC_50_: Half-maximal inhibitory concentration
