# Supplementary material for: A novel fatty acid metabolism-related signature identifies features of the tumor microenvironment and predicts clinical outcome in acute myeloid leukemia
Source: Lipids Health Dis. 2022 Aug 25;21:79. doi: 10.1186/s12944-022-01687-x (PMC9404605; doi:10.1186/s12944-022-01687-x)
Supplement: Supplementary file 1 — Additional file 1. [file 12944_2022_1687_MOESM1_ESM.docx]

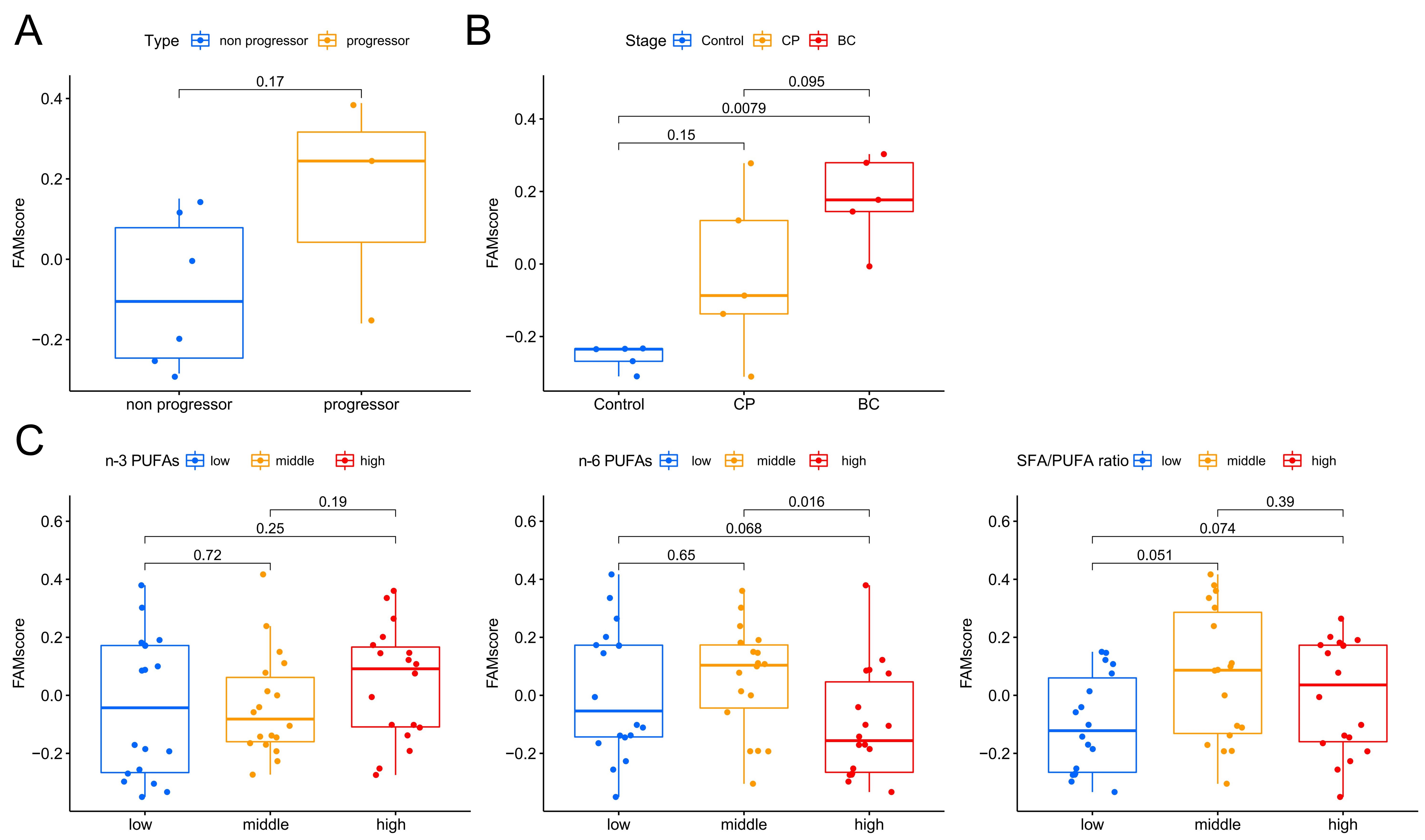


**Figure S1. Differences in FAMscore of AML patients with different treatment responses or CML patients in different stages and the relationship between FAMscore and fatty acid content in normal human peripheral blood.** (A) Differences in FAMscore of AML patients after ven/aza treatment. (B) Differences in FAMscore between normal people and patients with different stages of CML. CP: chronic phase; BC: blast crisis. (C) Differences in FAMscore of normal people with low, medium, and high-fatty acid content or ratios in peripheral blood, respectively. PUFA: polyunsaturated fatty acid; SFA: saturated fatty acid.


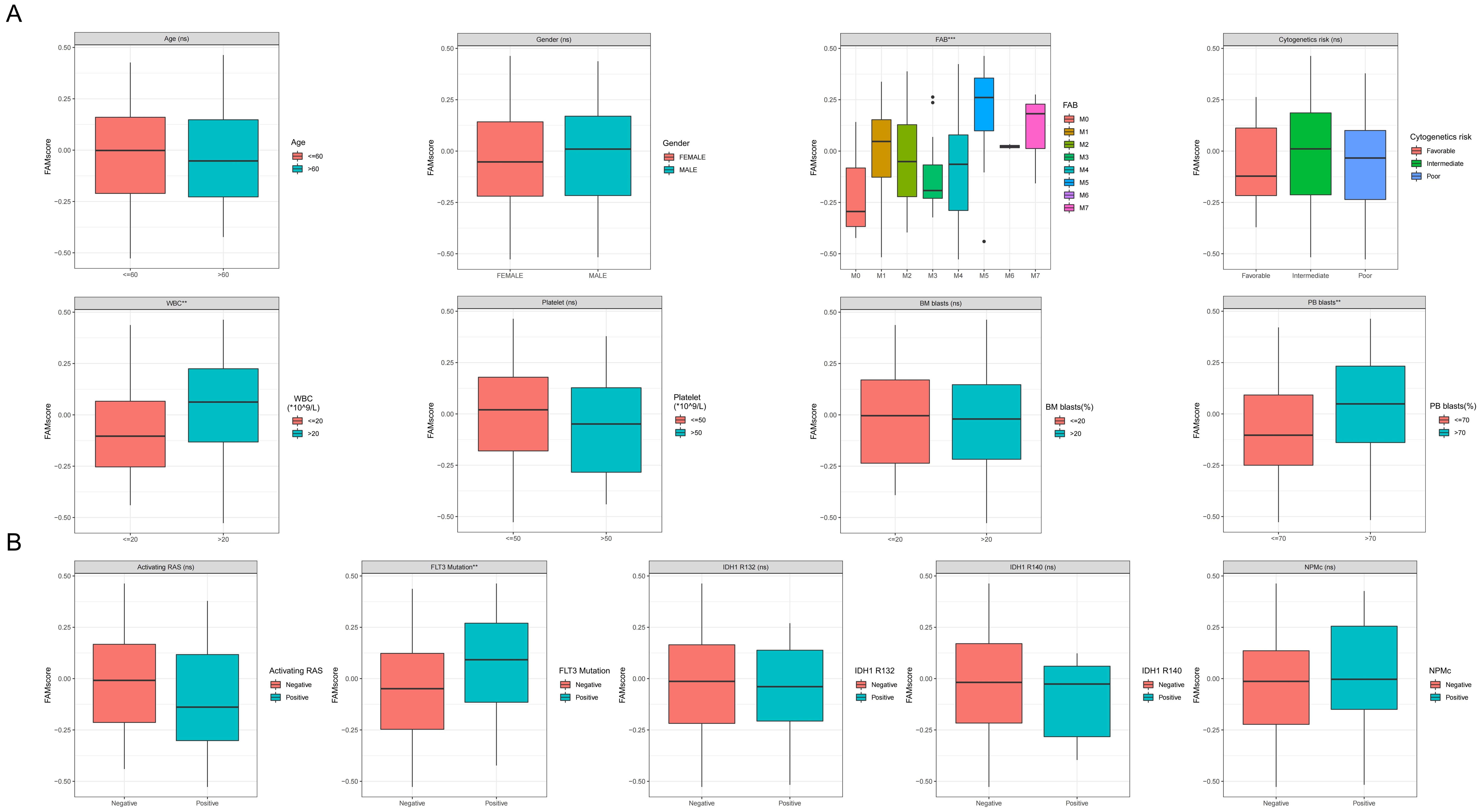


**Figure S2. Differences in FAMscore among patients with different clinicopathological characteristics.** (A) Differences in FAMscore for different individual characteristics, such as age, gender, French–American–British classification, cytogenetic risk, white blood cell or platelet count, and bone marrow or peripheral blood blast count. (B) Differences in FAMscore for different somatic variation signatures (e.g., RAS-activating, FLT3/IDH mutation, or cytoplasmic nucleophosmin profile). * *P* < 0.05; ** *P* < 0.01; * *P* < 0.001.


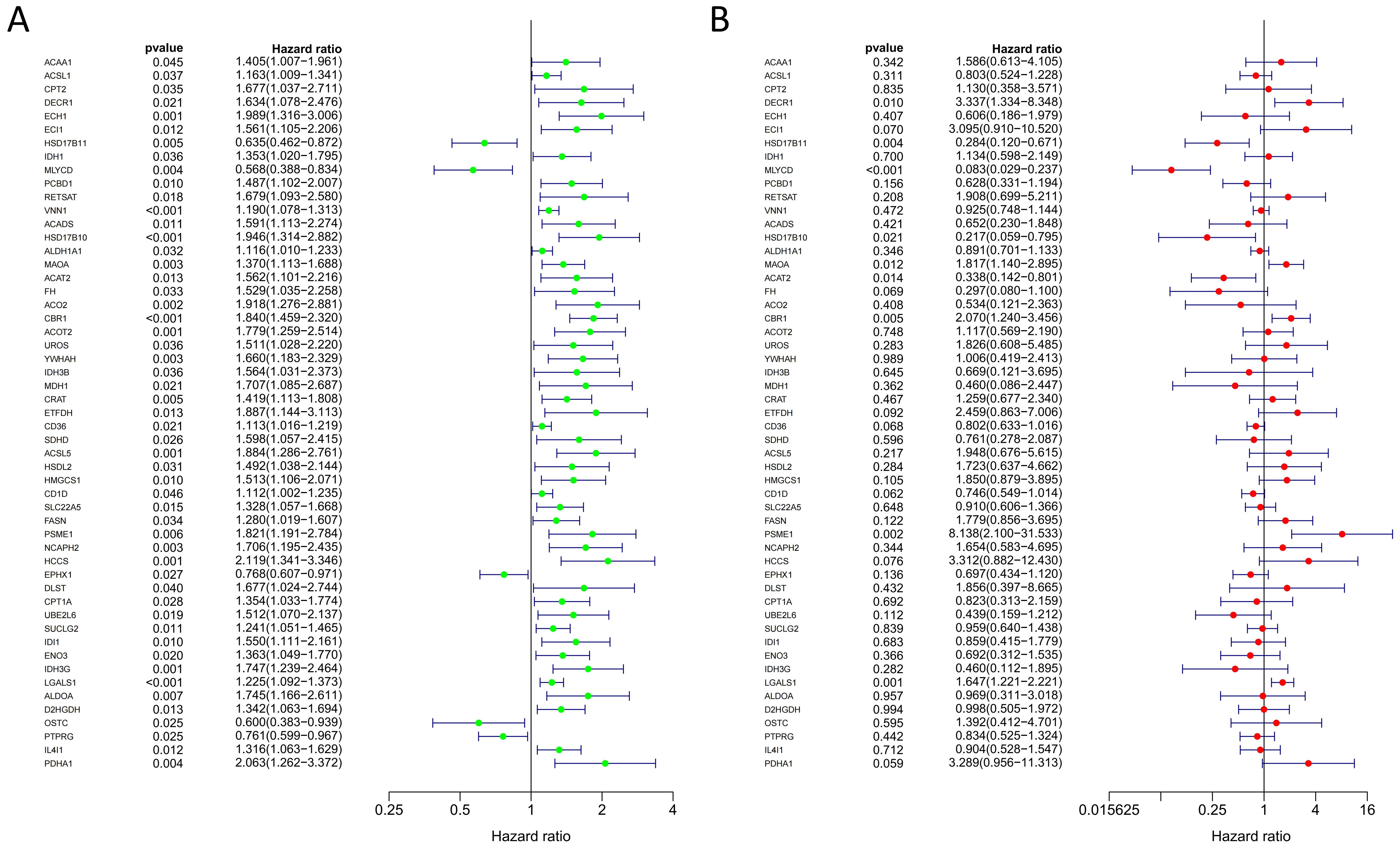


**Figure S3. Prognostic analysis of DEGs.** (A) Univariate cox regression analysis. (B) Multivariate cox regression analysis.


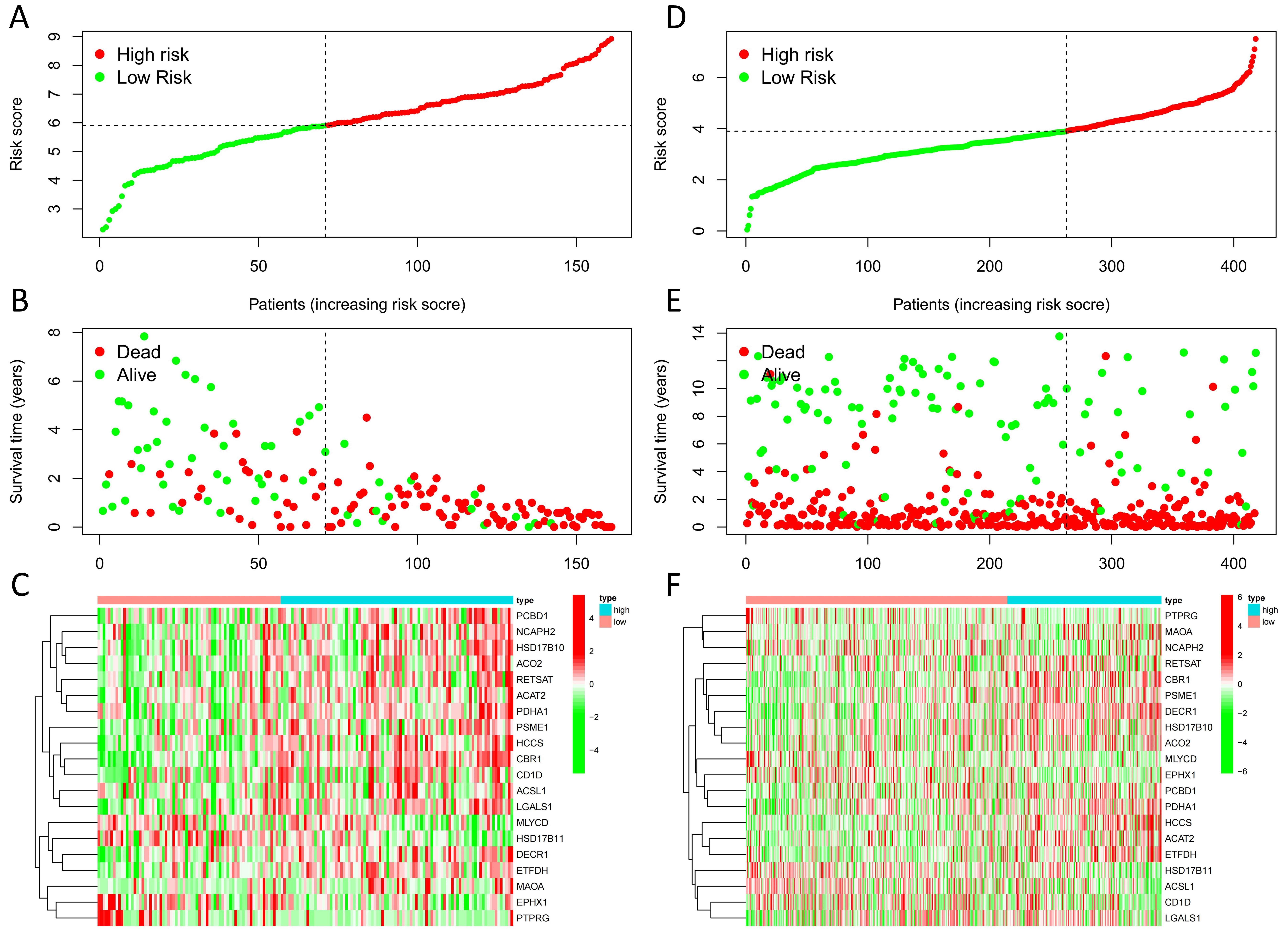


**Figure S4. The relationship between risk scores and survival status of samples in the training cohort and validation cohort.** (A-C) TCGA cohort. (D-F) GEO cohort. A and D: Distribution of risk scores ranked from low to high; B and E: Comparison of survival status between low- and high- risk score groups. C and F: Heatmap of 20 genes selected by LASSO Cox regression analysis.


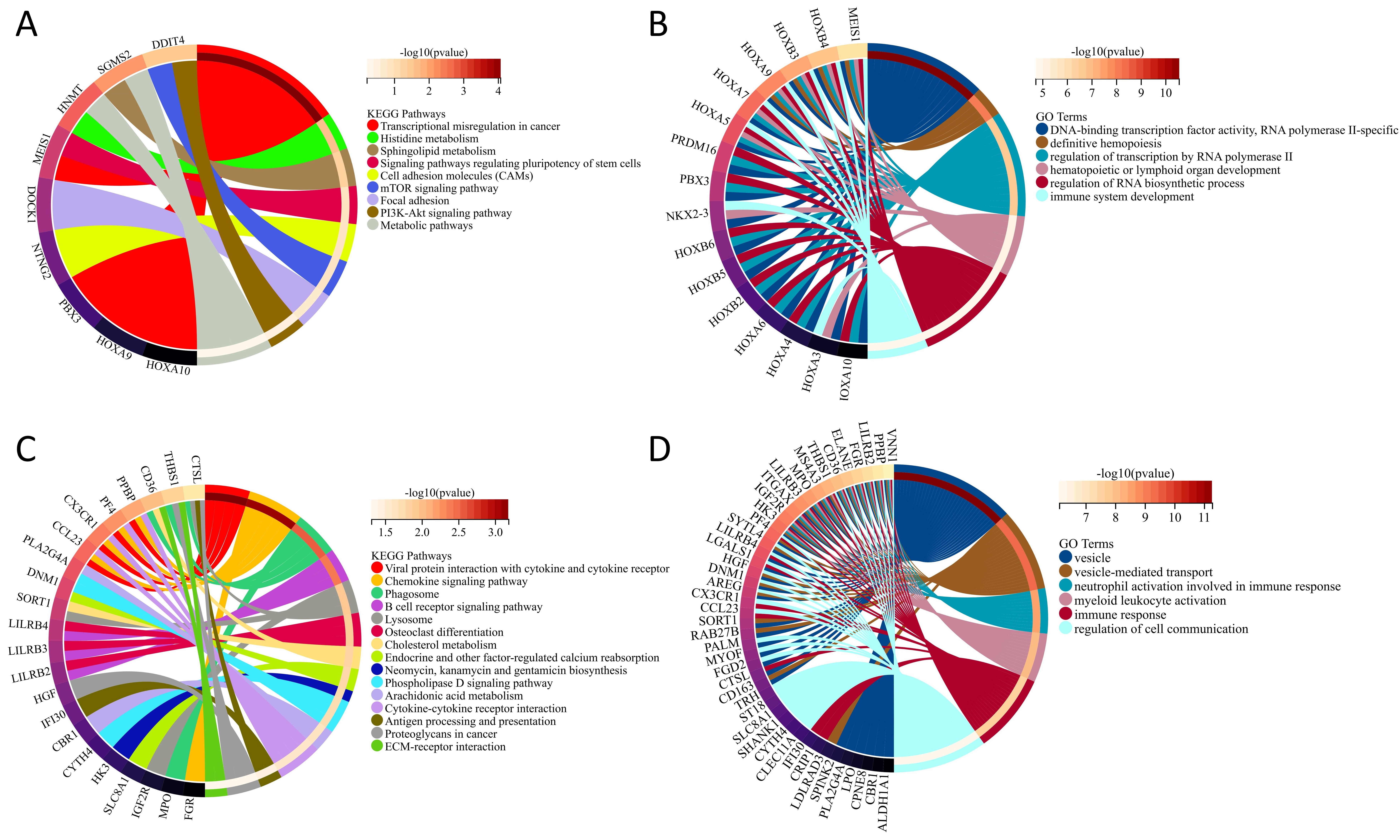


**Figure S5. Functional analysis of PPI sub networks.** (A-B) Green sub network. (C-D) Red sub network. A and C: KEGG enrichment analysis; B and D: GO annotation.
